# Graft and Patient Survival After Liver Transplantation for Primary Sclerosing Cholangitis: A French National Cohort Study

**DOI:** 10.1111/liv.70557

**Published:** 2026-03-12

**Authors:** Florian Veyre, Claire Francoz, Domitille Erard, Eleonora DeMartin, Laure Elkrief, Camille Besch, Olivier Boillot, Filomena Conti, Sébastien Dharancy, Christophe Duvoux, Jean Gugenheim, Jean Hardwigsen, Marie‐Noëlle Hilleret, Vincent Leroy, Isabelle Ollivier‐Hourmand, Marilyne Gratien, Pauline Houssel‐Debry, Nassim Kamar, Olivier Roux, Jean‐Baptiste Hiriart, Marie Irles‐Depe, Sophie Pellegrin, François Durand, Georges‐Philippe Pageaux, Stéphanie Faure, Audrey Coilly, Sylvie Radenne, Faouzi Saliba, Didier Samuel, Claire Vanlemmens, Marianne Latournerie, Karim Boudjema, Ephrem Salamé, Olivier Chazouillères, Christophe Corpechot, Jérôme Dumortier

**Affiliations:** ^1^ Service D'hépato‐Gastroentérologie, Centre de Référence Des Maladies Inflammatoires Des Voies Biliaires et Des hépatites Autoimmunes—Filière FILFOIE, Hôpital de la Croix‐Rousse Hospices Civils de Lyon Lyon France; ^2^ Université Claude‐Bernard Lyon 1 Lyon France; ^3^ Service D'Hépatologie et Transplantation Hépatique Hôpital Beaujon, APHP Clichy France; ^4^ Centre Hépato‐Biliaire, Hôpital Paul Brousse, AP‐HP Université Paris Saclay, Unité Inserm Villejuif France; ^5^ Service D'Hépato‐Gastroentérologie, Centre de Référence Des Maladies Vasculaires du Foie Hôpital Trousseau, CHU Tours Tours France; ^6^ Service de Chirurgie Hépato‐Bilio‐Pancréatique et Transplantation Hépatique CHRU Hautepierre Strasbourg France; ^7^ Fédération Des Spécialités Digestives, Centre de référence Des Maladies Inflammatoires Des Voies Biliaires et Des hépatites Autoimmunes—Filière FILFOIE, Hôpital Edouard Herriot Hospices Civils de Lyon Lyon France; ^8^ Service D'Hépatogastroentérologie, Unité Médicale de Transplantation Hépatique, Hôpital Pitié Salpêtrière, AP‐HP Sorbonne Université Paris France; ^9^ Service D'Hépatologie Hôpital Claude Huriez, CHRU Lille Lille France; ^10^ Service D'Hépatologie Hôpital Henri Mondor, APHP Créteil France; ^11^ Service de Chirurgie Digestive et de Transplantation Hépatique, Hôpital Universitaire de Nice Université de Nice‐Sophia‐Antipolis Nice France; ^12^ Service Chirurgie générale et Transplantation Hépatique Hôpital La Timone, APHM Marseille France; ^13^ Service D'hépato‐Gastroentérologie CHU Grenoble‐Alpes Grenoble France; ^14^ Service D'hépato‐Gastroentérologie Caen France; ^15^ Service D'Hépato‐Gastroentérologie et Nutrition Hôpital Dupuytren, CHU de Limoges Limoges France; ^16^ Service Des Maladies du Foie Hôpital Universitaire de Pontchaillou Rennes France; ^17^ Département de néphrologie et Transpantation D'organes, Hôpital Rangueil, CHU de Toulouse, Axe TImE, INSERM UMR 1291, Toulouse Institute for Infectious and Inflammatory Diseases (Infinity) Université Paul Sabatier Toulouse France; ^18^ Service D'Hépatologie et de Transplantation Hépatique Hôpital Haut Lévêque, CHU de Bordeaux Pessac France; ^19^ Département D'Hépatologie et Transplantation Hépatique CHU Saint Eloi Montpellier France; ^20^ Service D'Hépatologie et Soins Intensifs Digestifs Hôpital Jean Minjoz Besançon France; ^21^ Service D'hépato‐Gastroentérologie CHU Dijon‐Bourgogne Dijon France; ^22^ Service de Chirurgie hépato Biliaire et Digestive Hôpital universitaire de Pontchaillou Rennes France; ^23^ Service de Chirurgie Digestive, Oncologique et Endocrinienne, Transplantation Hépatique Hôpital Trousseau, CHU Tours Tours France; ^24^ Service D'Hépatologie, Hôpital Saint‐Antoine, APHP, Centre de Référence Des Maladies Inflammatoires Des Voies Biliaires et Des Hépatites Autoimmunes Filière FILFOIE Paris France

## Abstract

**Background:**

A significant proportion of patients presenting a primary sclerosing cholangitis (PSC) will require liver transplantation (LT). The present study aimed to investigate graft loss and patient death in a large cohort of patients.

**Methods:**

We conducted a nationwide multicenter retrospective study including all adult patients transplanted for PSC in France From 1985 to 2019.

**Results:**

Were included 571 patients; median follow‐up after LT was 89.0 months (IQR, 43.0–151.0). Patient survival at 5, 10 and 20 years after LT was 88.2%, 81.2% and 62.6%. After exclusion of patients who died during the first month after LT, 37 patients (6.6%) died during the first 2 years and the main cause was malignancies (*n* = 15, 40.5%, including 12 cases of recurrent cholangiocellular carcinoma). After 2 years, 90 patients (17.2%) died; the two main causes were malignancies (*n* = 36, 40.0%, including 13 cases of colorectal cancer) and sepsis (*n* = 23, 25.6%, of which 7 were related to recurrent PSC). Graft survival at 5, 10 and 20 years was 89.5%,78.7% and 62.7%. Independent factors associated with late patient death (after 2 years) were an older age at LT, a bilio‐digestive anastomosis and the use of preventive UDCA; independent factors associated with late graft loss were recurrent PSC, cellular rejection, a younger age at LT, and the use of tacrolimus (protective).

**Conclusions:**

Our results emphasise that the prognosis after LT for PSC could be improved by better detection of cholangiocellular carcinoma before LT, and colorectal cancer after LT.

## Lay Summary

Patient mid‐term (< 2 years) survival is impaired due to recurrence of cholangiocellular carcinoma, especially incidental cholangiocellular carcinoma. Patient long‐term survival is impaired due to colorectal cancer, for which screening (colonoscopy) is insufficient. Graft survival is impaired due to recurrence of primary sclerosing cholangitis, often requiring re‐transplantation.

**Abbreviations**
AIH: autoimmune hepatitis
ALP: alkaline phosphatase
ALT: alanine‐Amino‐Transferase
AST: aspartate‐Amino‐Transferase
AZA: Azathioprine
CCA: cholangiocellular carcinoma
CST: corticosteroid
CYA: cyclosporine
EVR: everolimus
HAT: hepatic artery thrombosis
HCC: hepatocellular carcinoma
IBD: inflammatory bowel disease
LT: liver transplantation
MMF: mycophenolate mofetil
PNF: primary non‐function
PSC: primary sclerosing cholangitis
PTLD: post‐transplant lymphoproliferative disorders
reLT: liver retransplantation
rPSC: primary sclerosing cholangitis recurrence
SIR: sirolimus
TAC: tacrolimus
UDCA: ursodeoxycholic acid

## 1 Introduction

Primary sclerosing cholangitis (PSC) is a chronic liver disease of unknown origin, characterised by extra‐ and/or intrahepatic bile duct inflammation leading to fibrosis and stenosis [1, 2]. There is no effective medical treatment to reduce the risk of biliary/liver complications, in particular secondary biliary cirrhosis, recurrent cholangitis episodes, cholangiocellular carcinoma (CCA) and hepatocellular carcinoma (HCC), so a significant proportion of patients will require liver transplantation (LT). This indication of LT remains rare; it accounts for approximately 5% of LT in France [3]. When compared to other indications/liver diseases, the median age at LT for PSC is lower, ranging from 31 to 49 years according to available studies [4]. PSC can recur in about 20%–25% of LT recipients, usually between 5 and 10 years after LT, and even up to 20 years after LT, with some differences between reports mainly because of heterogeneity in diagnosis definition and length of follow‐up [5].

The impact of the recurrence of PSC (rPSC) on graft survival is debated and appears to have a pejorative impact in many studies, even though only a few studies have looked specifically at graft survival in this specific indication [6]. Several studies focusing on rPSC risk factors have described a negative impact of rPSC on the graft, with a higher risk of liver retransplantation (reLT), compared to the recurrence of other liver autoimmune diseases, such as primary biliary cholangitis or autoimmune hepatitis [5, 6, 7]. The factors associated with graft survival have not been strongly identified by now. Similarly, patient survival after LT for PSC has been poorly studied. It seems to be very good, more than 90% at 5 years in several studies [5, 8, 9]. The causes of patient death are not well described in the literature. The main causes of early death include sepsis, CCA recurrence, and multiorgan failure; in the long term, the main causes are *de novo* malignancies and cardiovascular events [5, 6, 7, 8]. The impact of rPSC on overall survival is debated; some studies found a higher risk of patient death related to rPSC, but several studies described no impact due to frequent recourse to retransplantation [5, 7].

The aim of the present study was to investigate patient and graft survival after LT for PSC, in a large‐scale study involving all French LT centres over a long period of time, in order to present data on risk factors for death and graft loss, thereby enabling better management of these patients.

## 2 Patients and Methods

### 2.1 Study Population and Study Design

All patients aged 18 and older, who underwent a first LT for PSC in France from January 1985 to March 2019 were identified from the databases of the French Agence de la Biomédecine (ABM) and all French transplant centers. The diagnosis was established before LT according to the European Association for the Study of the Liver guidelines [10] and was then confirmed by a compatible histopathological examination of the explanted native liver. Patients with autoimmune hepatitis (AIH)‐PSC variants were included.

Clinical and biological data were collected from medical records. Treatment of PSC before LT was determined, including duration and dose of ursodeoxycholic acid (UDCA), endoscopic and radiological treatments. Complications of PSC were collected, including recurrent cholangitis, refractory pruritis, secondary biliary cirrhosis, CCA, HCC; severity of cirrhosis was assessed using MELD and CHILD scores. We also collected data on inflammatory bowel disease (IBD) at the inscription on the waiting list, including medical and surgical treatment history, IBD activity, and current treatments at the time of LT. Donor characteristics and donor/recipient mismatches in the HLA system were determined for the main loci A, B, DR, and DQ.

All patients received grafts from cadaveric or living donors. The initial immunosuppressive regimen was based on a calcineurin‐inhibitor: cyclosporine (CYA) or tacrolimus (TAC). Induction therapy by anti‐interleukin‐2 receptor antibodies was mainly administered in case of acute kidney injury. Other types of induction therapies (antithymocyte globulin, OKT3, daclizumab, intravenous immunoglobulins) were used infrequently. Starting on postoperative day 1, corticosteroid (CST) was tapered down to reach a maintenance dose of 0 to 5 mg/day at 6 months post‐transplantation. Azathioprine (AZA), mycophenolate mofetil (MMF), and sirolimus (SIR)/everolimus (EVR) were either administered as part of an initial triple immunosuppressive regimen or introduced during follow‐up as a maintenance immunosuppressive agent. Follow‐up visits were ensured every 3–12 months after the first year post‐LT. Occurrence of biliary and vascular complications, rejection episodes, CMV infections, cancers were systematically reported.

Diagnosis of rPSC was based on the Mayo Clinic's definition [11] which combines:

– A diagnosis of PSC prior to LT.
– Cholangiographic or histological signs > 90 days after LT.
– Exclusion of secondary cholangitis, in particular of ischemic or biliary origin.

Data collected at the time of rPSC included immunosuppressive regimen, biochemical parameters, IBD activity and treatment. Treatment adjustments after rPSC diagnosis were recorded, as well as its impact on the graft (bacterial cholangitis, evolution to cirrhosis). We defined preventive UDCA as a treatment with UDCA introduced within 1 month after LT, without any other indication than the aim to reduce the risk of rPSC.

The end of follow‐up corresponded to patient death, date of loss to follow‐up, or last medical examination.

We have separated the post‐LT period into three phases according to the specific issues highlighted during these phases, corresponding to: (1) the first‐month post‐LT (short‐term phase 1); (2) the first 2 years post‐LT excluding the first month (mid‐term phase 2); and (3) long‐term survival after the first 2 years (long‐term phase 3).

This study was conducted in accordance with the Declaration of Helsinki. According to French law (Loi Jardé), retrospective studies do not require Institutional Review Board (IRB) approval.

### 2.2 Statistical Analysis

Data were described in their totality and according to recurrence status using median with interquartile range (IQR) for continuous variables and number (percentage) for categorical variables. Categorical variables were compared with the Chi‐square or Fischer's exact tests and quantitative variables were compared using the Student *t*‐test or non‐parametric tests (Mann–Whitney or Kruskall‐Wallis tests) when appropriate. We used Younden's method to determine the relevant thresholds of quantitative variables for survival analyses. Centers were categorised as high‐ or low‐volume centers according to the median number of patients included in the study.

The two events of interest were patient death and graft loss. For graft survival, we chose reLT as an event, as well as patient deaths related to a hepatic cause. We divided the analysis into three periods of follow‐up after LT: early postoperative period (1 month), mid‐term (1 month–2 years), and long‐term (after 2 years). Survival curves were constructed using the Kaplan–Meier model and compared with the Cox test in univariate analysis. The Cox proportional hazards regression model was used in both univariate and multivariate models. To measure the impact of rPSC, we also used time‐dependent covariates in the Cox model. Results were expressed as hazard ratios (HRs) and confidence intervals (CI). All significant variables in the univariate analysis with a level set at *p* < 0.05 were included in the multivariate models, after selecting non‐collinear values. The validity of the multivariate model was tested, respecting the proportional hazards assumption (Shoenfeld residuals test). Statistical analyses were done using R software, version 4.2.1 (2022‐06‐23 ucrt).

## 3 Results

### 3.1 Study Population

The main characteristics of the 571 patients included in the study are shown in Table 1. The study population consisted of a majority of men (68.1%), with a median age at LT of 42.0 years (IQR, 32.0–52.8); 22.2% of LT were performed before the year 2000. One‐third of patients had a history of recurrent cholangitis episodes at the time of listing and 11.8% were diagnosed with an AIH‐PSC variant. Median follow‐up after LT was 89.0 months (IQR, 43.0–151.0; range, 0–413.1).

**TABLE 1:** Clinical and biological characteristics of the study population (*n* = 571).

Characteristics	*n* (%)	Number of missing data
	OR median [IQR]	
Gender (M/F)	389/182	0
Age at first LT (y)	42.0 [32.0; 52.8]	0
LT before year 2000	127/571 (22.2%)	0
*Before LT*		
Complications of PSC		
Recurrent cholangitis	147/475 (30.9%)	96
Refractory pruritis	55/423 (13.0%)	148
AIH/PSC variant	62/524 (11.8%)	47
Cirrhosis	318/490 (64.9%)	81
Complications of cirrhosis		
Ascites	118/318 (37.1%)	0
Digestive bleeding on portal hypertension	77/318 (24.2%)	0
Hepatic encephalopathy	25/318 (7.9%)	0
Portal thrombosis	23/318 (7.2%)	0
Medical treatments		
UDCA	349/401 (87.0%)	70
CST	48/416 (11.5%)	155
AZA	26/417 (6.2%)	154
Endoscopic/radiological treatments		
Endoscopic biliary prosthesis	61/432 (14.1%)	139
Transhepatic biliary drain	17/432 (3.9%)	139
Biology		
Bilirubin (μmol/l)	67.5 [26.0; 160.0]	85
yGT (U/l)	226 [109.0; 465.0]	192
ALP (U/l)	395 [218.0–650.0]	199
ACE (ng/l)	1.60 [1.0; 2.4]	402
CA19‐9 (U/mL)	27.9 [9.8; 74.3]	382
MELD	14.2 [9.4; 18.3]	120
IBD	301/531 (56.7%)	40
UC	191/301 (63.5%)	0
CD	97/301 (32.2%)	0
Indeterminate colitis	13/301 (4.3%)	0
*At time of LT*		
Donor age (years)	43.0 [26.0; 57.0]	125
Gender mismatch donor/recipient	180/434 (41.5%)	137
CMV D+/R‐ mismatch status	185/394 (32.4%)	177
High‐volume center	443/571 (77.6%)	0
LT		
Cold ischemia time (minutes)	480 [375; 608]	102
LT Surgery duration (minutes)	420 [330; 494]	283
Biliary anastomosis (n and %): bilio‐digestive/bilio‐biliary	396/492 (80.5%)	79
1985–1989	8/0 (100%/0%)	10
1990–1999	75/5 (93.75%/6.25%)	29
2000–2004	67/14 (82.7%/17.3%)	14
2005–2009	56/21 (72.7%/27.3%)	15
2010–2014	112/16 (87.5%/12.5%)	8
2015–2019	77/40 (65.8%/34.2%)	3
Right liver transplant	30/537 (5.6%)	34
Number of red blood cells concentrate perfused	2.0 [0; 4.0]	165
CCA		
History of CCA before LT	11	0
CCA on explant	27	0
Incidental*	22	0
Known before LT	5	0
HCC		
History of HCC before LT	8	0
HCC on explant	12	0
Incidental	7	0
Known before LT	5	0
Induction treatment	83/531 (15.6%)	40
Polyclonal antibodies	13	
Anti IL2R	70	
Initial immunosuppressive treatment		
CYA	104/551 (18.9%)	20
TAC	447/551 (81.1%)	20
AZA	98/540 (18.1%)	31
MMF	362/539 (67.2%)	32
CST	536/548 (97.8%)	23
*After LT*		
Preventive treatment with UDCA	111/468 (23.7%)	133
UDCA at last follow‐up	228/458 (49.8%)	143
rPSC	141/544 (25.9%)	27
Immunosuppressive treatment at last follow‐up		
CYA	55/493 (11.2%)	78
TAC	385/494 (77.9%)	77
AZA	40/493 (8.1%)	78
MMF	237/491 (48.3%)	76
CST	280/490 (54.9%)	75
EVR or SIR	64/493 (13.0%)	78
Malignancies		
Non cutaneous solid organ malignancies		
De novo CRC	26/533 (4.9%)	38
Post‐LT recurrent CRC	1/533 (0.2%)	38
de novo CCA	2/533 (0.4%)	38
Post‐LT recurrent CCA	18/533 (3.4%)	38
Kidney	9/533 (1.7%)	38
Lung	5/533 (0.9%)	38
Pancreas	3/533 (0.6%)	38
de novo HCC	1/533 (0.2%)	38
Post‐LT recurrent HCC	1/533 (0.2%)	38
Other	27/533 (5.6%)	38
PTLD	28/462 (6.1%)	109
Skin cancer	32/533 (6.0%)	38
Basal cell carcinoma	20/32	
Squamous cell carcinoma	10/32	
Both	2/32	
Melanoma	1	
Graft loss		
Phase 1 (< 1 month after LT)	15	
Phase 2 (> 1 month to < 2 years after LT)	20	
Phase 3 (> 2 years after LT)	93	
Patient death		
Phase 1 (< 1 month after LT)	11	
Phase 2 (> 1 month to < 2 years after LT)	37	
Phase 3 (> 2 years after LT)	90	

*Note:* Data shown as either *n* (%) or median (interquartile range) for continuous variables. Percentage of incidental CCA over total number of LT, by 5‐year period.

Abbreviations: AZA, azathioprine; CD, Crohn's disease; CST, corticosteroids; CYA, cyclosporine A; EVR, everolimus; IBD, inflammatory bowel disease; LT, liver transplantation; MMF, mycophenolate mofetil; ns, not significant; PTLD, post transplantation lymphoma disease; rPSC, recurrence of PSC; SIR, sirolimus; TAC, tacrolimus; UC, ulcerative colitis; UDCA, ursodeoxycholic acid.
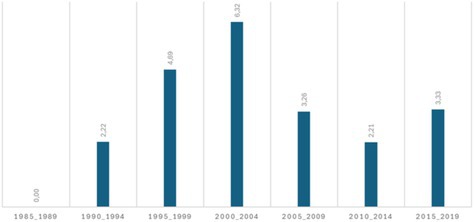

Initial immunosuppressive treatment (within the first year after LT) consisted of TAC for 81.1% of patients and CYA for 18.9% of patients. In 85.3% of cases, an antimetabolite was also administered and CST in almost all patients (97.8%).

One hundred and forty‐one patients (25.9%) developed rPSC during follow‐up. The cumulative rate of rPSC gradually increased with time, with a median delay of 59.0 months (IQR, 28.0–107.0; range, 5.0–347.0) after LT. Cumulative incidence of rPSC at 5, 10, 15, 20, and 25 years after LT was 15.6%, 27.8%, 37.9%, 48.3% and 52.6%, respectively. Median time between rCSP and graft loss was 40.5 months.

**FIGURE 1:**
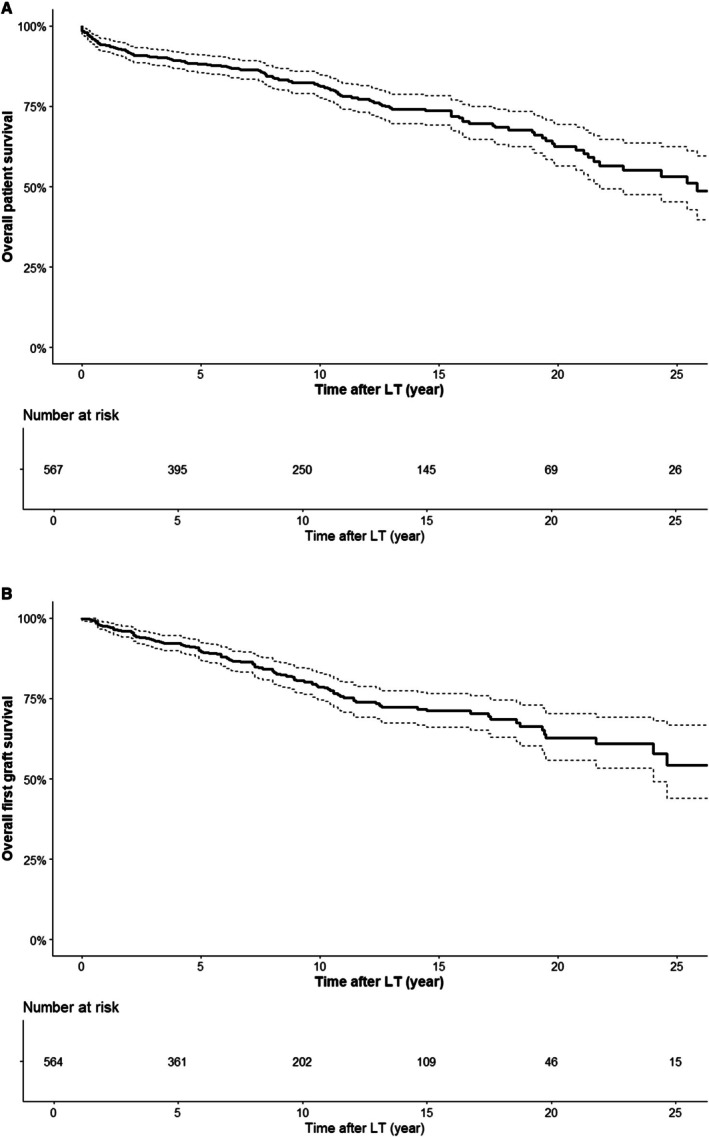
Overall patient and first graft survival. (A) Overall patient survival. Patient survival at 5, 10, and 20 years after LT were 88.2%, 81.2%, and 62.6% respectively. (B) Overall first graft survival. First graft survival at 5, 10, and 20 years after LT were 89.5%, 78.7%, and 62.7% respectively.

During follow‐up after LT, 138 patients died (24.2%); overall patient median survival was 321.0 months (95% CI 261.0; NA) and median time to death was 74.5 months (95% CI 46.0; 97.0). Figure 1A shows the overall patient survival curve after LT. Patient survival at 5, 10 and 20 years after LT was 88.2%, 81.2% and 62.6%, respectively. Regarding graft survival, 128 first grafts were lost (22.4%); the first graft median survival was 369.0 months. Figure 1B shows the first graft overall survival curve after LT. First graft survival at 5, 10 and 20 years after LT was 89.5%,78.7% and 62.7%, respectively.

### 3.2 Early Survival

Within the first month after the LT (i.e., Phase 1), 11 patients (1.9%) died; the causes of death are described in Table 2. The main causes were sepsis (*n* = 3, 27.3%), postoperative haemorrhage (*n* = 2, 18.2%) and PNF (*n* = 2, 18.2%).

**TABLE 2:** Causes of patient death according to post‐LT period.

	Phase 1 (first month post‐LT) (11 deaths)	Phase 2 (First 2 years post‐LT excluding the first month) (37 deaths)	Phase 3 (After the first 2 years post‐LT) (90 deaths)
Sepsis	3 (27.3%)	11 (29.7%)	16 (18.2%)
Postoperative haemorrhage	2 (18.2%)	0	0
First graft PNF	2 (18.2%)	0	0
Malignancy	0	15 (40.5%) 12 CCA 1 recurrence of CRC 1 pancreatic cancer 1 lung cancer	36 (39.3%) 13 CRC 8 CCA 3 lung cancers 2 pancreatic cancers 9 PTLD
HAT	0	2 (5.4%)	0
rPSC consequences	0	0	9 (10.2%), among whom 7 deaths from infectious cholangitis 2 deaths from secondary biliary cirrhosis
Cardiovascular	0	0	5 (5.7%)
Other/unknown	4 (36.4%)	9 (24.3%)	24 (27.3%)

*Note:* Data shown as *n* (%).

Abbreviations: CCA, cholangiocarcinoma; CRC, colorectal cancer; HAT, hepatic artery thrombosis; LT, liver transplantation; PNF, primary non function; PTLD, post‐transplant lymphoproliferative disorder; rPSC, recurrence of primary sclerosing cholangitis.

During the first month after LT, we observed 15 losses of the first graft. The two causes of the first graft loss were hepatic artery thrombosis (HAT; *n* = 12, 80.0%) and primary non‐function (PNF; *n* = 3, 20.0%). Of these 15 graft losses, 13 patients (86.7%) underwent an early reLT; the other 2 patients died (2 PNF).

### 3.3 Mid‐Term Survival

After the exclusion of patients who died during the first month after LT, 37 patients (6.6%) died during the first 2 years after the first LT. The causes of patient death are described in Table 2. The two main causes were malignancies (*n* = 15, 40.5%) and sepsis (*n* = 11, 29.7%). Of the 15 cancer cases, 12 were CCA. The three other cancers were 1 recurrence of CRC operated 2 years before LT, 1 pancreatic cancer, and 1 lung cancer. These 15 patients died within a median of 18.0 months (IQR, 6.0–22.5) after LT.

**FIGURE 2:**
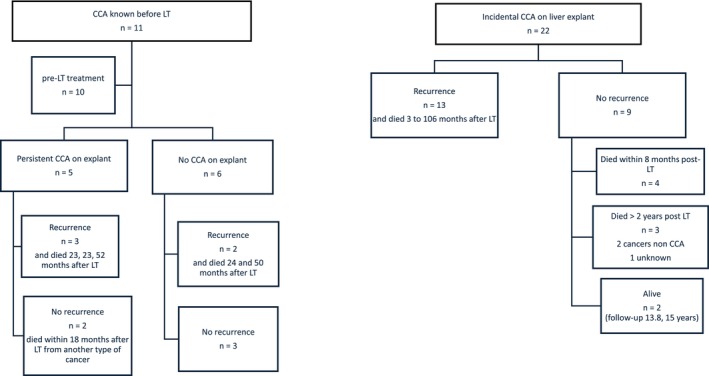
Outcome of patients with CCA (recurrence and survival).

Regarding CCA, 1 patient had been treated before LT and considered cured (this was a case of gallbladder cancer, which had only been treated surgically), two patients presented a previously known CCA and both had undergone medical treatment before LT (chemotherapy and radiotherapy), and the other nine cases were discovered incidentally on the explanted liver. The years of LT of patients with diagnosis of incidental CCA on explant ranged from 1985 to 2017. Biliary dysplasia lesions on prior cholecystectomy material were reported for two patients who had incidental CCA discovered on the liver explant. Figure 2 describes the outcome of all patients with history of CCA at the time of LT. In case of incidental CCA, the mortality rate was 20/22 (90.9%), including 13/22 (59.0%) related to CCA recurrence, 4/22 (18.2%) due to early mortality within the first 8 months, and 2/22 (9.1%) related to another cancer, or 1/22 (4.5%) of unknown cause more than 2 years after LT. Median survival rate after CCA recurrence (all patients died) was 6.4 months (95% CI 1.9–13.4).

After the exclusion of graft losses during the first‐month post‐LT, 20 patients lost their first graft before 2 years post‐LT, of whom 17 (85.0%) underwent a reLT and 3 (15.0%) died of a hepatic cause without having reLT before. The main causes of first graft loss during this period were HAT (*n* = 13, 65.0%) and chronic rejection (*n* = 5, 25.0%); one patient died because of haemorrhage and for the last patient, the cause of death remained unknown.

### 3.4 Late Patient Survival

During late follow‐up, after excluding patients who died during the first 2 years after LT, 90 patients (17.2%) died. Cumulative incidence of late patient death was 6.1%, 13.6%, 21.7%, 33.4% at 5, 10, 15, 20 years after LT, respectively. The causes of death are described in Table 2. The two main causes were malignancies (*n* = 36, 40.0%) and sepsis (*n* = 23, 25.6%, of which 7 were sepsis from cholangitis secondary to rPSC). Table 3 describes the population according to whether they died > 24 months or not, and the significant risk factors of death > 24 months.

**TABLE 3:** Risk factors for late patient death (> 2 years).

	No death	Death > 24 months	Univariate analysis		Multivariate analysis	
	*N = 433*	*N = 90*	*p*	HR (95% CI)	*p*	HR (95% CI)
Median age at LT	41.0 [31.0; 52.0]	46.0 [33.0; 54.0]	0.001	1.03 [1.01; 1.05]		
Age > 42 at LT	193 (44.5%)	53 (58.9%)	< 0.001	2.29 [1.48; 3.55]	< 0.001	2.65 [1.48; 4.76]
Age > 50 at LT	120 (27.7%)	36 (40.4%)	< 0.001	2.48 [1.60; 3.84]		
Male gender	286 (66.1%)	64 (71.1%)	ns			
Comorbidities at inscription on waiting list						
Diabetes	22 (5.1%)	7 (10.0%)	0.016	2.65 [1.20; 5.84]		
HTA	34 (7.9%)	7 (10.3%)	ns			
Dyslipidemia	19 (4.4%)	5 (8.2%)	0.07	2.34 [0.93; 5.91]		
LT before 2000	63 (14.5%)	43 (47.8%)	ns			
Bilio‐digestive anastomosis	301 (69.5%)	64 (95.6%)	0.043	3.32 [1.04; 10.6]	0.034	3.59 [1.10; 11.72]
High‐volume center^a^	301 (69.5%)	73 (81.1%)	ns			
Immunosuppressive regimen after LT						
Induction	66 (15.2%)	10 (12.7%)	ns			
CYA	62 (14.3%)	36 (43.4%)	ns			
TAC	363 (83.8%)	50 (61.0%)	ns			
AZA	62 (14.3%)	28 (35.4%)	ns			
MMF	307 (70.9%)	32 (39.5%)	ns			
SIR	3 (0.7%)	1 (1.2%)	ns			
CST	411 (94.9%)	79 (97.5%)	ns			
Preventive treatment by UDCA	81 (18.7%)	22 (33.3%)	0.02	1.83 [1.09; 3.07]	0.033	1.83 [1.05; 3.20]
rPSC	107 (24.7%)	33 (42.3%)	ns			
Rejection	129 (29.7%)	39 (52.7%)	ns			
Post‐LT IBD	280 (64.7% 67.0%)	52 (70.3%)	ns			
Known before LT	232 (82.9%)	43 (82.4%)	ns			
Diagnosed after LT	48 (16.1%)	9 (17.6%)	ns			

*Note:* Data shown as either *n* (%) or median (interquartile range) for continuous variables. Each factor was tested in univariate analysis by logrank test. A level set at *p* < 0.05 was chosen to select variables for the multivariate analysis.

Abbreviations: AZA, azathioprine; CST, corticosteroids; CYA, cyclosporine A; IBD, inflammatory bowel disease; LT, liver transplantation; MMF, mycophenolate mofetil; ns, not significant; rPSC, recurrence of PSC; TAC, tacrolimus; UDCA: ursodeoxycholic acid.

^a^ Threshold = 21.

Regarding cancer mortality, the causes of death included CRC (*n* = 13/36, 36.1%), CCA (*n* = 8/36, 22.2%), lung cancer (*n* = 3/36, 8.3%), pancreatic cancer (*n* = 2/36, 5.6%), and PTLD (post‐transplant lymphoproliferative disorder—*n* = 9/36, 25.0%).

More specifically, regarding CRC mortality, 27 patients developed colorectal cancer during follow‐up (5.2%), among whom 1 was a metastatic recurrence of a cancer treated before LT. Of these patients, 13 died, including the patient with post‐TL recurrence (HR = 2.61 [1.41;4.81], *p* < 0.001). Regarding *de novo* CRC, the median rate of available colonoscopy was 0.29/patient and per year, in the IBD population.

In this follow‐up period > 2 years post‐LT, 8 patients presented a CCA. Of these patients, six developed a recurrence of CCA, later than the majority of patients in our cohort (since the majority of CCA recurrences occurred during the first 2 years); all these 6 patients died (HR = 29.7, 95% CI [11.1;79.3]) (Figure 2). In addition, two patients presented a *de novo* CCA; these patients had no history of pre‐LT CCA and no CCA on the explant; they had all undergone biliodigestive anastomosis. These 2 patients also died from CCA.

Two patients presented HCC. One of the two cases was a metastatic recurrence of a known HCC before LT, treated by chemoembolization before LT, present on the liver explant; the recurrence occurred 2 years after surgery and was a metastatic recurrence leading to systemic treatment. The second case was a *de novo* HCC in a patient with no history of HCC and no HCC on the explant; the patient was too impaired to receive treatment and died.

Finally, 32 patients (6.0%) developed non‐melanoma skin cancers, with no impact on mortality.

Table 3 describes the risk factors of patient death > 2 years. The factors associated with patient death > 2 years were older age at LT (in particular age > 42 was a risk factor with HR = 2.29 [1.48;3.55], *p* < 0.001), diabetes at inscription on waiting list (HR = 2.65 [1.20;5.84], *p* = 0.016), bilio‐digestive anastomosis (3.32 [1.04;10.6], *p* = 0.043), and preventive treatment with UDCA (1.85 [1.10;3.12], *p* = 0.02). Dyslipidemia at inscription on waiting list was a risk factor at the limit of statistical significance (HR = 2.34 [0.93;5.91], *p* = 0.07).

The year of LT had no statistical impact on patient survival, nor did the type of initial immunosuppressive regimen. Finally, rPSC (considered or not as a time‐dependent variable) and rejection events had no impact on patient survival.

Preventive UDCA was initially prescribed in 111 patients, with a median dose of 14.2 mg/kg/day. In 15 patients (13.5%) a high dose (> 20 mg/kg/day) was prescribed, with a median dose‐weight of 23.1 mg/kg/day. In the subgroup of patients with preventive UDCA ≤ 20 mg/kg/day, median dose‐weight was 13.7 mg/kg/day. Among patients treated with high dose preventive UDCA, risk of late death was significantly higher compared to small dose (*p* = 0.05). In the overall population, risk of late death was similar between patients who did not receive preventive UDCA and patients treated with dose of preventive UDCA ≤ 20 mg/kg/day, but higher in patients with high dose preventive UDCA > 20 mg/kg/day (*p* = 0.02) (Figure S1). Table S1 describes the patients who received, or not, preventive UDCA, and survived more than 2 years.

**FIGURE 3:**
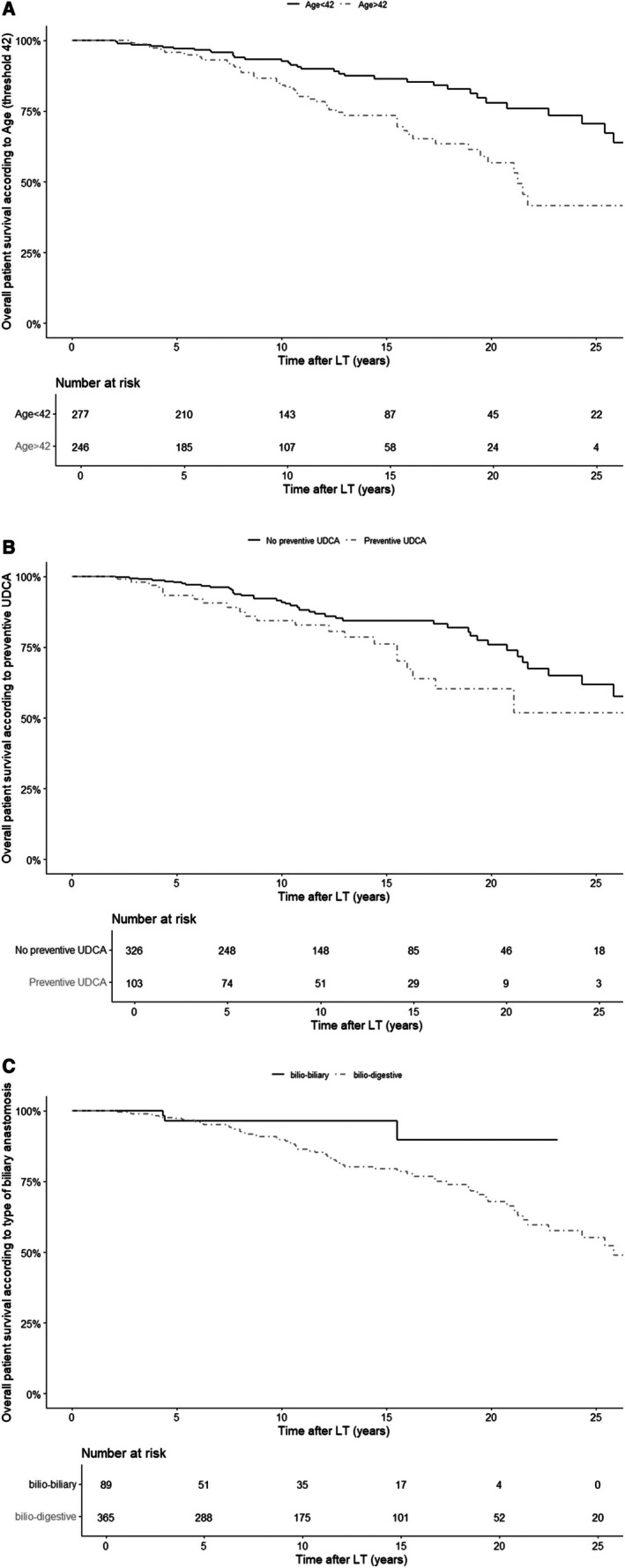
Risk factors of patient death > 2 years after LT. (A) Late patient survival curve according to age at LT, with a threshold at 42. Patient survival at 5, 10, and 20 years after LT were 97.1%, 92.7%, and 78.0% respectively in the group of patients <42 at LT and 95.8%, 84.2%, and 56.8% respectively in the group of patients > 42 at LT. (B) Late patient survival curve according to prescription of preventive UDCA. Patient survival at 5, 10 and 20 years after LT were 93.2%, 84.4% and 60.4% respectively in the group with preventive UDCA and 97.9%, 90.9% and 75.6% respectively in the group without preventive UDCA. (C) Late patient survival curve according to biliary anastomosis type. Patient survival at 5, 10 and 20 years after LT were 96.5%, 96.5% and 89.6% respectively in the group of patients with bilio‐biliary anastomosis at LT and 97.3%, 89.8% and 67.8% respectively in the group of patients with bilio‐digestive anastomosis at LT.

In multivariate analysis, factors associated with patient death were an older age at LT (in particular > 42 (median) with HR = 2.65 [1.48;4.76] *p* < 0.001), bilio‐digestive anastomosis (HR = 3.59 [1.10;11.72], *p* = 0.034), and preventive UDCA (HR = 1.83 [1.05;3.20], *p* = 0.032). Figure 3 illustrates the impact of all independent prognostic factors.

### 3.5 Late Graft Survival

After excluding graft loss during the first 2 years, 83 patients were reLT, the main cause of reLT was rPSC (*n* = 62, 75.0%). Among these 83 patients, 23 patients died after reLT. Furthermore, 10 patients died of hepatic causes without reLT, of whom 7 died of sepsis due to cholangitis, 2 from the progression of cirrhosis related to rPSC, and 1 from the progression of cirrhosis unrelated to rPSC. Cumulative incidence of late graft loss was respectively 6.6%, 18.3%, 26.1%, 34.9% at 5, 10, 15, 20 years after LT.

Table 4 describes the risk factors of first graft loss > 2 years post‐LT. In univariate analysis, the risk factors of first graft loss were young age at LT (age at LT < 42, *p* = 0.001, HR = 1.99, 95% CI [1.30;3.05]), LT before 2000 (*p* = 0.035, HR = 1.60, 95% CI [1.03;2.49]), use of CYA (*p* = 0.002, HR = 2.04, 95% CI [1.31;3.17]), rPSC (*p* < 0.001, HR = 6.73, 95% CI [4.14;11.0]), an episode of acute cell rejection (*p* < 0.001, HR = 2.49 95% CI [1.61;3.83]) and the presence of IBD after LT (*p* = 0.025, HR = 1.72, 95% CI [1.07;2.78]); use of TAC was a protective factor (*p* = 0.006, HR = 0.53, 95% CI [0.34;0.83]).

**TABLE 4:** Risk factors for first graft loss (> 2 years).

	No first graft loss	First graft loss	Univariate analysis		Multivariate analysis	
	*N = 388*	*N = 93*	*p*	HR (95% CI)	*p*	HR (95% CI)
Median age at LT	44.0 [33.0; 54.0]	36.0 [27.0; 45.0]	< 0.001	0.96 [0.95; 0.98]	0.028	0.98 [0.96; 0.99]
Age at LT < 60	340 (87.6%)	91 (97.8%)	0.016	4.74 [1.17; 19.3]		
Age at LT < 42	164 (42.3%)	59 (63.4%)	0.001	1.99 [1.30; 3.05]	0.048	1.60 [1.01; 2.53]
Sex male	257 (66.2%)	65 (69.9%)	ns			
LT before 2000	61 (15.7%)	41 (44.1%)	0.035	1.60 [1.03; 2.49]		
Bilio‐digestive anastomosis	270 (79.2%)	74 (90.2%)	ns			
High‐volume center^a^	298 (76.8%)	69 (74.2%)	ns			
Immunosuppressive regimen after LT						
Induction	61 (16.4%)	7 (8.75%)	ns			
CYA	54 (14.2%)	39 (44.3%)	0.002	2.04 [1.31; 3.17]		
TAC	330 (87.3%)	53 (60.2%)	0.006	0.53 [0.34; 0.83]		
AZA	54 (14.4%)	32 (37.6%)	0.072	1.53 [0.96; 2.45]		
MMF	273 (72.8%)	37 (43.5%)	ns			
CST	370 (98.1%)	85 (98.8%)	ns			
Preventive UDCA	81 (25.2%)	13 (17.8%)	ns			
rPSC	69 (18.5%)	71 (77.2%)	< 0.001	6.73 [4.14; 11.0]	< 0.0001	5.51 [3.34; 9.10]
Cellular rejection	104 (28.3%)	53 (60.9%)	< 0.001	2.49 [1.61; 3.83]	< 0.001	2.41 [1.54; 3.77]
Post‐LT IBD	237 (64.1%)	65 (73.9%)	0.025	1.72 [1.07; 2.78]		

*Note:* Data shown as either *n* (%) or median (interquartile range) for continuous variables. Each factor was tested in univariate analysis by logrank test. A level set at *p* < 0.05 was chosen to select variables for the multivariate analysis.

Abbreviations: AZA, azathioprine; CST, corticosteroids; CYA, cyclosporine A; IBD, inflammatory bowel disease; LT, liver transplantation; MMF, mycophenolate mofetil; ns, not significant; rPSC, recurrence of PSC; TAC, tacrolimus; UDCA, ursodeoxycholic acid.

^a^ Threshold = 21.

**FIGURE 4:**
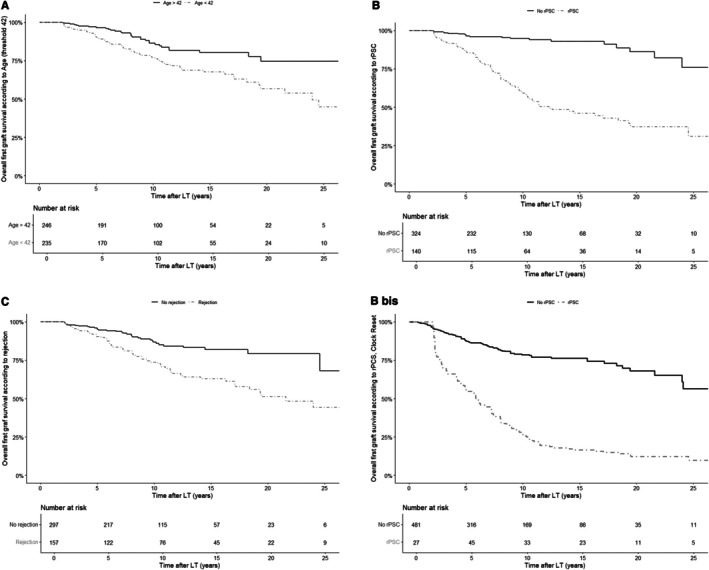
Risk factors of first graft loss > 2 years after LT. (A) First graft late survival curve according to age at LT, with a threshold at 42. First graft survival at 5, 10, and 20 years after LT were respectively 96.5%, 86.4%, and 74.6% in the group of patients > 42 at LT and respectively 90.1%, 76.8%, and 56.7% in the group of patients < 42 at LT. 4B. First graft late survival curve according to rPSC.First graft survival at 5, 10, and 20 years after LT were respectively 96.4%, 94.7%, and 86.1% in the group without rPSC and respectively 86.2%, 59.2%, and 37.2% in the group with rPSC. (B) bis. First graft late survival curve according to rPSC (with time‐dependent covariate modelling). First graft survival at 5, 10, and 20 years after LT were respectively 87.4%, 78.6%, and 68.1% in the group without rPSC and respectively 55.9%, 26.5%, and 12.3% in the group with rPSC, with time‐dependent covariate in Cox model. (C) First graft late survival curve according to acute cellular rejection. First graft survival at 5, 10, and 20 years after LT were respectively 95.1%, 86.7%, and 79.3% in the group without acute cellular rejection and respectively 90.5%, 73.7%, and 51.3% in the group with acute cellular rejection.

In multivariate analysis, using a standard Cox model, factors associated with first graft loss were rPSC (HR = 5.51, [3.34;9.10], *p* < 0.0001), cellular rejection (HR = 2.41 [1.54;3.77], *p* < 0.001), and a younger age at LT (in particular < 42 (median) with HR = 1.60 [1.01;2.53], *p* = 0.048 or age as a continuous variable HR = 0.98 [0.96;0.99], *p* = 0.028). By using a time dependant model with rPSC as time dependent covariate, factors associated with first graft loss were rPSC (HR = 4.89 [3.46;6.91], *p* < 0.0001), cellular rejection (HR = 2.52 [1.79;3.54], *p* < 0.001), a younger age at LT (in particular < 42 (median) with HR = 1.81 [1.28;2.56], *p* < 0.001), whereas TAC was protective (HR = 0.53 [0.37;0.75], *p* < 0.001). Figure 4 illustrates the impact of significant independent factors influencing graft survival in both models.

## 4 Discussion

PSC is a relatively rare indication for LT, accounting for around 5% of all LT in adults in France. This proportion has remained stable over the past few decades. Because of the clinical characteristics of the patients, it is a singular indication in comparison to frequent indications such as alcohol‐related liver disease, metabolic syndrome, or viral hepatitis, and even in comparison with other autoimmune liver diseases, HAI and PBC. This explains why outcomes after LT in this particular population have certain specificities. Patients suffering from PSC are at greater risk of various cancers, both hepatic (including CCA, HCC, and gallbladder cancer) and extrahepatic (including CRC, when PSC is associated with IBD, and probably pancreatic adenocarcinoma) [12, 13, 14]. There is currently no treatment that can modify the natural history of the disease, and many patients require LT [2, 7]. Our study is the largest retrospective cohort study to look specifically at survival after LT for PSC, based on individual data. We schematically distinguished three periods after LT, according to the causes of death and graft loss. We confirm herein that patient survival is good (62.6% at 20 years) but impaired by malignancies, in particular pre‐transplant CCA and *de novo* colorectal cancer. In contrast, graft survival (62.7% at 20 years) is reduced because of rPSC, which often requires reLT, sometimes multiple. We acknowledge that our study has some limitations. First, this study is retrospective in nature, and some data were not available for all patients. Second, the long study period, which is a strength in terms of length of follow‐up and number of patients included, may also lead to heterogeneity. Indeed management of patients significantly evolved during these past 4 decades.

One of the particularities of LT for PSC is the risk of early postoperative sepsis, since these patients may have presented recurrent episodes of cholangitis, which can lead to the indication of LT [2, 15]. Multidrug‐resistant sepsis, secondary to previous antibiotic treatments, may also be a concern. Nevertheless, septic complications were not frequent causes of death in the early postoperative period (first month) in our population. It can be assumed that the majority of patients with episodes of bacterial cholangitis before transplantation are transplanted at a time when infection is controlled by antibiotic therapy. In very rare cases, transplantation may be the last resort for non‐controllable infections. Outside sepsis, the other causes of early death or graft loss seem not to be specific to the indication of PSC and are usual, often related to surgical complications.

Our experience strongly confirms that mid‐term prognosis after LT for PSC is impaired by the presence of CCA. There are two distinct situations. First, in recent years, limited perihilar CCA became an indication for LT, in selected cases, and after a neoadjuvant chemoradiation therapy (Mayo protocol, year 2000) [16, 17, 18]. In this context, patient survival is acceptable. The second situation is that of incidental diagnosis of CCA on the liver explant, and the prognosis is therefore very poor (only 2 out of 22 patients survived without recurrence from our 22 cases). We report here such cases, from 1985 to 2017, suggesting that it remains a current issue. Safdar and coll. studied the impact of an incidental diagnosis of CCA on the explant in a cohort of 95 patients, 58 of whom had PSC; this study demonstrated a lower survival rate in this situation, of 34.4% at 5 years. During the first 2 years after LT, after the exclusion of very early deaths during the first month, 37 patients died (6.6%) and the main cause of death was cancer, with CCA in the first place (*n* = 12, i.e., 32.4% of causes of death), of which 75% (*n* = 9) were discovered incidentally on the liver explant. Cambridge and coll. published in 2021 the results of a meta‐analysis including 20 studies and 428 patients [19]. No randomised control trial is available. The pooled 1‐, 3‐ and 5‐year overall survival rates after LT without neoadjuvant therapy were 71.2% (95% CI 62.2%–79.4%), 48.0% (95% CI 35.0%–60.9%), and 31.6% (95% CI 23.1%–40.7%). These improved to 82.8% (95% CI 73.0%–90.8%), 65.5% (95% CI 48.7%–80.5%), and 65.1% (95% CI 55.1%–74.5%) if neoadjuvant chemoradiation was completed [19]. Pooled recurrence after 3 years was 24.1% (95% CI 17.9%–30.9%) with neoadjuvant chemoradiation, 51.7% (95% CI 33.8%–69.4%) without, supporting that neoadjuvant chemoradiation confers long‐term survival in highly selected patients able to complete neoadjuvant chemoradiation followed by transplantation. PSC patients appeared to have the most favourable outcomes. Therefore, the detection of CCA in patients with PSC remains a major goal. There are no guidelines on the screening and monitoring of dysplasia in patients with PSC. Many experts recommend regular monitoring using imaging, mostly MRI, and CA19‐9 [13, 15]. Endoscopic retrograde cholangiopancreatography with biliary biopsies is another strategy, although the sensitivity of cytology is low [13]. Furthermore, high‐grade dysplasia is a prelude to developing CCA but LT for biliary dysplasia is debated, especially as between 20% and 57% of patients with pre‐LT dysplasia do not end up with cancer on the explant [20]. The very low survival rate in this context of incidental diagnosis of CCA encourages a systematic screening as exhaustive as possible.

*De novo* malignancy occurs more commonly after LT than in the general population [21, 22]. The overall mortality rate from *de novo* malignancy in this patient population is high [1, 2, 3, 5]. Indeed, *de novo* malignancy is one of the leading causes of late mortality in LT recipients [4, 6, 7, 8]. Variable incidence rates for *de novo* malignancy (2%–16%) have been reported in the literature, but vary depending on the source of information, type of malignancies, geographical origin, main indication for LT, length of follow‐up or era of transplantation [3, 9, 11, 12, 13]. Watt and coll. analysed the National Institute of Diabetes and Digestive and Kidney Diseases' liver transplantation database of 798 adults who received transplants from 1990 to 1994 and long‐term follow‐up data through January 2003. In this patient population, 171 adult patients developed 271 *de novo* malignancies. The probability of developing any nonskin malignancy was highest in patients with initial PSC (22% at 10 years) or alcohol‐related liver disease (18% at 10 years); all other recipients had a 10% probability. The probabilities of death after diagnosis of post‐transplant lymphoma disease (PTLD) and solid malignancy were 44.0% and 38.0% at 1 year and 57.6% and 53.1% at 5 years, respectively. Our results are in line with these results. The most relevant goal is undoubtedly the screening for colorectal cancer in patients with IBD. Annual surveillance colonoscopy in the post‐LT period is recommended for PSC‐IBD patients subset given their high risk for colorectal cancer. We report here a low median rate of available colonoscopy, 0.29/patient and per year, in the IBD population. Although the recommendation for annual colonoscopy is more recent than the start of our study period, it covers the vast majority of it. We believe it is unlikely that a significant number of examinations were omitted during individual data procurement, as patients are usually followed up in the transplant centre. Mouchli and coll. from the Mayo Clinic in Rochester aimed to determine the cumulative incidence of/risk factors for long‐term cancer‐related mortality in patients with PSC after LT from 293 adult patients who underwent a LT for PSC without CCA from 1984 to 2012, with follow‐up through 2015 [23]. Over a median time of 11.5 years after LT, 64 patients (21.8%) developed 73 nonskin cancers, including 46 solid‐organ cancers (renal, 11; colorectal, 11; prostate, 7; breast, 5; pancreas, 5; ovarian/endometrial/vulvar cancers, 3; and de novo CCA, 4). Twenty‐two patients developed hematologic malignancies (posttransplant lymphoproliferative diseases, 18; Hodgkin disease, 2; and myelodysplastic syndrome, 2). Five patients developed melanoma. This strongly recalls our findings, that PSC patients develop a large spectrum of malignancies, in addition to colorectal cancers. The 1‐, 5‐, 10‐, and 20‐year cumulative incidences of cancer were 2.1%, 8.6%, 18.7%, and 27%, respectively. The mortality of patients with PSC who developed cancer was higher than that of patients with PSC without cancer (HR 2.2; *p* < 0.01). On multivariate analysis, the recipient's age and elevated pre‐LT INR were associated with an increased risk of *de novo* nonskin malignancy. Outside the endoscopic screening, a specific strategy for all *de novo* malignancies screening could probably be discussed in this specific population.

In our study, we identified three risk factors of patient death late (> 2 years) after LT. The first risk factor is a higher age at 1st LT and needs no discussion. Not surprisingly, bilio‐digestive anastomosis was also a risk factor. This type of anastomosis has long been recommended to avoid a theoretical risk of long‐term complications by leaving a part of the native bile duct potentially diseased. However, several studies have highlighted the negative impact of this type of anastomosis, with a risk of reflux cholangitis, non‐anastomotic stenosis and early patient and graft survival [24, 25]. Pandanaboyana and coll. concluded their meta‐analysis that there was no disadvantage to bilio‐biliary anastomosis, and that this option could be considered in selected patients, notably after discussion with the surgical team, in patients without dominant stenosis and without dysplasia of the common bile duct [26, 27]. More surprisingly, we found that preventive UDCA was a risk factor of late patient death. UDCA is frequently used for the treatment of PSC outside LT and usual dose is 13–20 mg/kg/day. This frequently improves liver biochemical parameters but evidence that it influences natural history, in particular LT‐free survival, cirrhosis‐free survival or risk of death, is lacking. Furthermore, there is currently no evidence that this treatment can limit the risk of rPSC after LT [28], unlike PBC [29]. Higher doses have therefore been tested in several studies, to influence PSC natural history, again without efficacy. Moreover, some studies showed a higher risk of adverse events, whether compared to placebo or to the conventional dose of UDCA [30, 31, 32]. Our study found similar data, with the use of preventive UDCA found to be a significant and independent risk factor of death. Analysis of the specific subgroup of patients for whom preventive UDCA was prescribed revealed that 13.5% of them received doses > 20 mg/kg/day, which conferred an increased risk of death compared with patients for whom a dose ≤ 20 mg/kg/day had been prescribed. It is also important to note that preventive UDCA was not prescribed in all centres in France, so there may be a centre effect that could explain this effect of UDCA on mortality risk.

The major impact of rPSC on graft survival is strongly confirmed herein in our French population, as it was before from large multicentre studies in the United Kingdom, Germany, and the United States [8, 9, 12] which included long‐term clinical course outcomes after LT and reported both rates of rPSC and graft failures [6, 33, 34] which were comparable to our current study. We report an estimated actuarial risk for developing rPSC of 15.6%, 37.9%, and 52.6%, 5, 15, and 25 years after LT, respectively [28]; rPSC had a major impact on graft survival (it was the cause of ¾ of late graft loss) but not on patient survival because of a high rate of reLT. Hildebrang and coll. analysed 335 patients who underwent LT for PSC in 10 German transplant centers (1990–2006). The 1‐, 5‐, and 10‐year recipient and graft survival was 90.7%, 84.8%, 79.4% and 79.1%, 69.0%, 62.4%, respectively. rPSC was diagnosed in 20.3%. From the United States cohort of LT recipients for PSC, Gordon and coll. compared 241 living donor LT (LDLT) recipients to 65 deceased donor LT (DDLT) recipients transplanted between 1998 and 2013. The overall probability of rPSC was 22.4% at 10 years after LT, without significantly different for DDLT vs. LDLT recipients. For patients with rPSC, the 5‐year unadjusted patient and graft survival probabilities were 66.5% and 56.8%, respectively. Significant risk factors for graft failure included time‐dependent rPSC, time‐dependent biliary complication, CCA diagnosed at or before transplantation, laboratory MELD at transplant, and donor age. Significant risk factors for death included time‐dependent rPSC, time‐dependent biliary complications, CCA diagnosed at or before transplantation, laboratory MELD at transplant, and donor age.

In conclusion, our results from a large cohort with long‐term follow‐up emphasise that the prognosis after LT for PSC can be considered good. Nevertheless, according to the young age of these patients, the prognosis could be improved by better detection of CCA before LT and colorectal cancer after LT. The impact of rPSC on patient survival is very limited because of a high rate of reLT, possibly multiple.

## Author Contributions

F.V. collected the data and performed data analysis. D.E. performed data analysis. J.D. coordinated project contributions and supervised data analysis. F.V., O.C., C.C. and J.D. drafted the manuscript. C.F., E.d‐M., L.E., C.B., O.B., F.C., S.D., C.D., J.G., J.H., M.‐N.H., V.L., I.O.‐H., M.G., P.H.‐D., N.K., O.R., J.‐B.H., M.I.‐D., S.P., F.D., G.‐P.P., S.F., A.C., S.R., F.S., D.S., C.V., M.L., K.B., E.S., C.C. were responsible for and oversaw data collection. All authors revised and approved the final version of the manuscript.

## Funding

This work was supported by Filfoie, Bourse Filfoie 2020.

## Ethics Statement

This study was conducted in accordance with the Declaration of Helsinki.

## Consent

According to French law (Loi Jardé), retrospective studies do not require Institutional Review Board (IRB) approval.

## Conflicts of Interest

The authors declare no conflicts of interest.

## Supporting information


**Figure S1:** Late patient survival curve according to prescription and dose of preventive UDCA. Patient survival at 5, 10 and 20 years after LT were 92.1%, 88.5% and 66.0% respectively in the group with preventive UDCA with median dose ≤ 20 mg/kg/day, 100.0%, 60.6% and 30.3% respectively in the group with preventive UDCA with median dose > 20 mg/kg/day, and 97.9%, 90.9% and 75.6% respectively in the group without preventive UDCA.

**Table S1:** Comparison of patients who received, or not, preventive UDCA, and who survived more than 2 years.

## Data Availability

The data that support the findings of this study are available on request from the corresponding author. The data are not publicly available due to privacy or ethical restrictions.
