# Supplementary figures and images for: Graft and Patient Survival After Liver Transplantation for Primary Sclerosing Cholangitis: A French National Cohort Study
Source: Liver Int. 2026 Mar 12;46(4):e70557. doi: 10.1111/liv.70557 (PMC12980461; doi:10.1111/liv.70557)

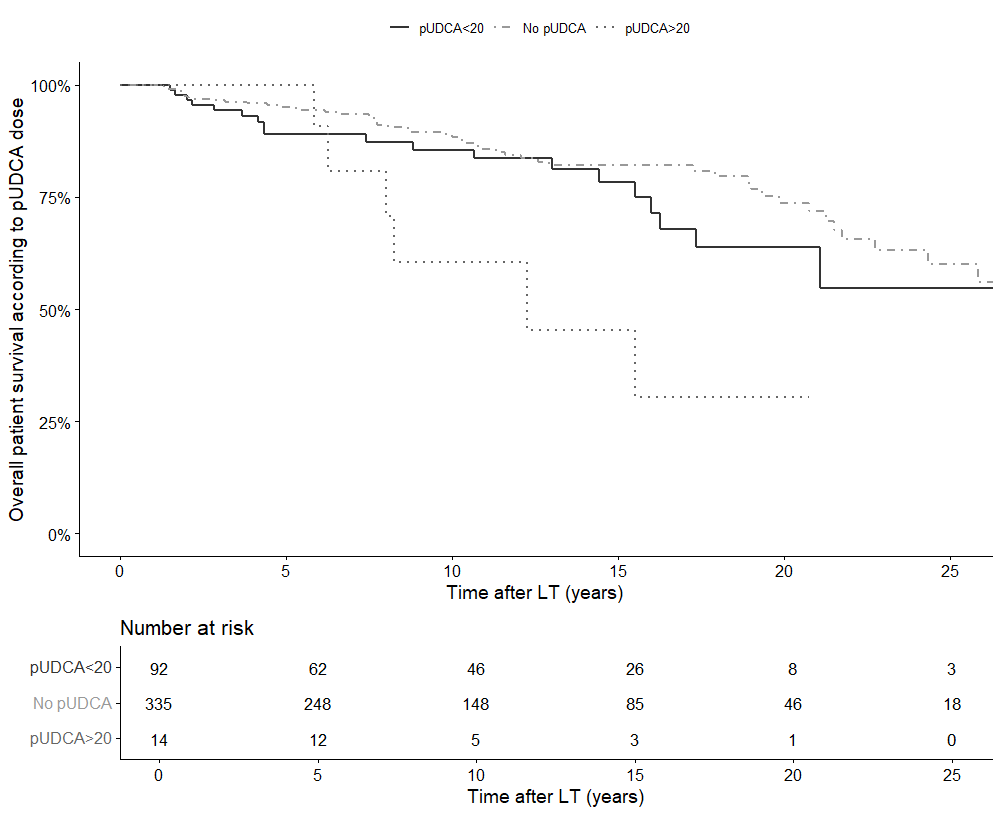

Supplement: Supplementary file 1 — Figure S1: Late patient survival curve according to prescription and dose of preventive UDCA. Patient survival at 5, 10 and 20 years after LT were 92.1%, 88.5% and 66.0% respectively in the group with preventive UDCA with median dose ≤ 20 mg/kg/day, 100.0%, 60.6% and 30.3% respectively in the group with preventive UDCA with median dose > 20 mg/kg/day, and 97.9%, 90.9% and 75.6% respectively in the group without preventive UDCA. [file LIV-46-0-s001.tiff]
