# Supplementary material for: Graft and Patient Survival After Liver Transplantation for Primary Sclerosing Cholangitis: A French National Cohort Study
Source: Liver Int. 2026 Mar 12;46(4):e70557. doi: 10.1111/liv.70557 (PMC12980461; doi:10.1111/liv.70557)
Supplement: Supplementary file 2 — Table S1: Comparison of patients who received, or not, preventive UDCA, and who survived more than 2 years. [file LIV-46-0-s002.docx]

**Supplementary Table 1. Comparison of patients who received, or not, preventive UDCA, and who survived more than 2 years**

|  | **No preventive UDCA**  **n = 326** | **Preventive UDCA**  **n = 103** | **p** |
| --- | --- | --- | --- |
| Age at first LT | 41.0 [31.0;52.0] | 43.0 [32.5;52.5] | 0.556 |
| Age at first LT > 42 | 149 (45.7%) | 52 (50.5%) | 0.463 |
| Male gender | 217 (66.6%) | 67 (65.0%) | 0.870 |
| HTA before LT  Dyslipidemia before LT  Diabetes before LT | 28 (9.2%)  14 (4.6%)  18 (6.0%) | 7 (6.8%)  10 (9.7%)  5 (4.9%) | 0.587  0.097  0.80 |
| LT before 2000 | 65 (19.9%) | 11 (10.7%) | 0.046 |
| Bilio-digestive anastomosis | 242 (83.2%) | 75 (75.0%) | 0.099 |
| Incidental CCA | 6 (1.9%) | 3 (2.9%) | 0.151 |
| Immunosuppressive regimen after LT  CYA  TAC  AZA  MMF  CST | 67 (20.7%)  263 (81.4%)  58 (18.0%)  214 (66.9%)  314 (97.8%) | 10 (9.71%)  92 (90.2%)  11 (10.8%)  77 (76.2%)  101 (99.0%) | 0.017  0.054  0.117  0.099  0.686 |
| rPSC | 82 (25.6%) | 23 (22.3%) | 0.588 |
| Rejection | 104 (32.8%) | 39 (38.2%) | 0.376 |
| Post-LT IBD | 219 (68.7%) | 62 (60.2%) | 0.144 |
